# Extracellular vesicle-delivered hsa_circ_0090081 regulated by EIF4A3 enhances gastric cancer tumorigenesis

**DOI:** 10.1186/s13008-024-00123-z

**Published:** 2024-06-11

**Authors:** Yanjie Mou, Kun Lv

**Affiliations:** Department of Tradition Chinese Medicine, Wuhan Third Hospital (Tongren Hospital of Wuhan University), No. 241, Pengliuyang Road, Wuchang District, Wuhan, 430060 Hubei China

**Keywords:** EIF4A3, EV, hsa_circ_0090081, Gastric cancer

## Abstract

**Background:**

Circular RNA (circRNA) and extracellular vesicles (EVs) in tumors are crucial for the malignant phenotype of tumor cells. Nevertheless, the mechanisms and clinical effects of EV-delivered hsa_circ_0090081 in gastric cancer (GC) are unclear. This study aimed to reveal the effect of eukaryotic translation initiation factor 4A3 (EIF4A3)-mediated hsa_circ_0090081 expression and EV-delivered hsa_circ_0090081 on GC progression.

**Methods:**

qRT-PCR was conducted to clarify hsa_circ_0090081 and EIF4A3 levels in GC tissues. Transmission electronic microscopy (TEM), nanoparticle tracking analysis (NTA), and Western blotting identified the EVs isolated from GC cells by ultracentrifugation. The roles of hsa_circ_0090081, EIF4A3, and EV-delivered hsa_circ_0090081 in GC cells were analyzed using Transwell, EdU, and CCK-8 assays. The regulatory role between EIF4A3 and hsa_circ_0090081 was investigated using RIP, qRT-PCR, and Pearson’s analysis.

**Results:**

Our study showed that hsa_circ_0090081 and EIF4A3 were highly expressed in GC, and hsa_circ_0090081 was associated with poor prognosis. Data revealed that hsa_circ_0090081 inhibition restrained GC cell proliferation, invasion, and migration. Additionally, EIF4A3 could bind to the pre-mRNA of PHEX (linear form of hsa_circ_0090081) to enhance hsa_circ_0090081 expression in GC cells. Moreover, EIF4A3 overexpression nullified the malignant phenotypic suppression caused by hsa_circ_0090081 silencing in GC cells. Furthermore, EVs secreted by GC cells delivered hsa_circ_0090081 to facilitate the malignant progression of targeted GC cells.

**Conclusion:**

This study showed that hsa_circ_0090081 was enhanced by EIF4A3 to play a promotive role in GC development. The results may help understand the mechanism of EIF4A3 and EV-delivered hsa_circ_0090081 and offer a valuable GC therapeutic target.

**Supplementary Information:**

The online version contains supplementary material available at 10.1186/s13008-024-00123-z.

## Background

Gastric cancer (GC) is among the five most prevalent cancers worldwide, causing more than 750,000 deaths in 2020 [1]. The etiology and pathogenesis of GC have not been determined. Although many causative factors have been identified, primary prevention remains challenging [2]. Recently, with the advancement of medical and surgical techniques, the understanding of GC pathogenesis has been continuously deepened, and the development of preventive methods and targeted therapies for GC has increased [3, 4]. Therefore, this study aimed to provide a valuable theoretical basis for GC-targeted therapy.

Circular RNA (circRNA) has no protein-coding potential in eukaryotic genomes and is widely expressed in various cell types [5]. With advancements in next-generation sequencing technologies, circRNAs have been found to occupy specific tissues and developmental stages and are closely associated with GC tumor development [6, 7]. Chen et al. [8] found elevated circDLG1 levels in GC tissues and distant metastatic lesions and reported poor prognosis in patients with aggressive tumor phenotypes. Lei et al. [9] found that overexpressed circCUL2 accelerated cancer progression by inhibiting cisplatin resistance and increasing autophagy in GC cells in vitro. Moreover, hsa_circ_0090081 is located at chrX:22,231,020–22266478, with the gene symbol PHEX, and has not been studied in human diseases. Therefore, the function and mechanisms of hsa_circ_0090081 in the GC process should be urgently studied.

Eukaryotic translation initiation factor 4A3 (EIF4A3) is a nuclear matrix protein and exon junction complex component belonging to the EIF4A protein family [10]. EIF4A3 can be closely connected with malignant tumors, such as prostate [11], breast [12], and cervical cancers [13]. In addition, EIF4A3 is highly regulated in GC, and its upregulation promotes cancer cell survival and metastasis [14]. However, the mechanism of EIF4A3 and hsa_circ_0090081 in GC has not been explored.

Extracellular vesicles (EVs) with 40–160 nm (mean, 100 nm) in diameter are enveloped in a lipid bilayer and secreted by most eukaryotic cells [15]. Its biology depends on its biologically active substances, such as metabolites, proteins, nucleic acids, and lipids, which could be transferred into target cells [16]. EV transport of circRNA has been studied for GC treatment. Xie et al. [17] showed that EV-delivered circSHKBP1 levels were significantly reduced in patients with GC postoperatively, and EVs with circSHKBP1 upregulation promoted GC cell angiogenesis and invasion. Therefore, we will investigate whether EV-delivered hsa_circ_0090081 is regulated by EIF4A3 to influence GC progression.

In this paper, we initially explored the differential level and biological function of hsa_circ_0090081 in GC. We aimed to reveal that EV-loaded hsa_circ_0090081 is positively regulated by EIF4A3 to accelerate GC oncogenicity. Our results will provide useful insights into the intrinsic mechanisms of GC onset and progression, as well as potentially curative targets.

## Results

### Upregulated hsa_circ_0090081 in GC predicts a worse survival prognosis

**Fig. 1:**
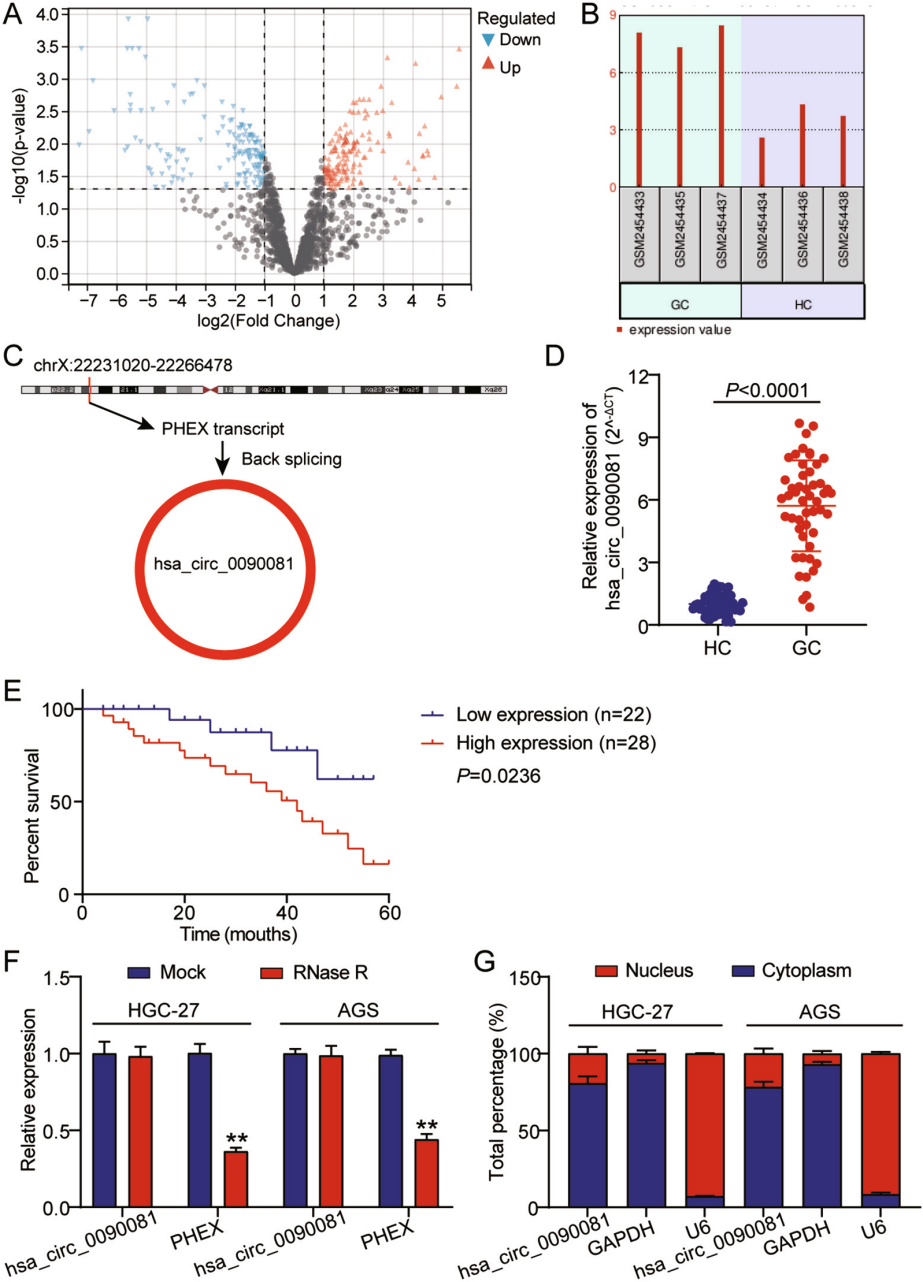
Upregulated hsa_circ_0090081 in GC predicts a worse survival prognosis. **A** Differentially expressed circRNAs in GSE93541, including three GC samples and three healthy control samples. **B** The levels of hsa_circ_0090081 in GC samples are based on GSE93541 data. **C** Bioinformatics analysis revealed the construction of hsa_circ_0090081. **D** qRT-PCR showed hsa_circ_0090081 levels in human GC and normal samples. **E** Analysis of the correlation between hsa_circ_0090081 expression and prognosis of patients with GC. **F** hsa_circ_0090081 and PHEX (linear of hsa_circ_0090081) levels in AGS and HGC-27 cells after RNase R digestion. **P < 0.001. **G** hsa_circ_0090081 level in the nucleus and cytoplasm of AGS and HGC-27 cells were revealed via cell nucleus/cytoplasm fraction isolation

GSE93541 is a circRNA expression microarray from a public database GEO DataSets including three GC samples and three healthy control samples, and the differentially expressed circRNAs were stored (Fig. 1A). Moreover, hsa_circ_0090081 was enriched in GC samples based on data from GSE93541 (Fig. 1B). In addition, bioinformatics analysis revealed that hsa_circ_0090081 was formed by the PHEX gene (Fig. 1C). Moreover, the hsa_circ_0090081 levels of 40 GC tissues and paired normal adjacent tissues were evaluated using qRT-PCR. Results showed that hsa_circ_0090081 levels were > fivefold in GC than in normal samples (Fig. 1D). Survival assessment further demonstrated that hsa_circ_0090081 upregulation could predict decreased survival time in patients with GC (Fig. 1E). Additionally, hsa_circ_0090081 showed RNase R resistance, whereas PHEX (linear of hsa_circ_0090081) showed RNase R sensitivity in AGS and HGC-27 cells after RNase R digestion (Fig. 1F). Subsequently, hsa_circ_0090081 characteristics were examined in GC cells. Analysis of nuclear and cytoplasmic fractions showed a predominantly cytoplasmic-enriched hsa_circ_0090081 (Fig. 1G), indicating hsa_circ_0090081 overexpression in GC and worse survival prognosis.

### hsa_circ_0090081 silencing curbs GC cells

**Fig. 2:**
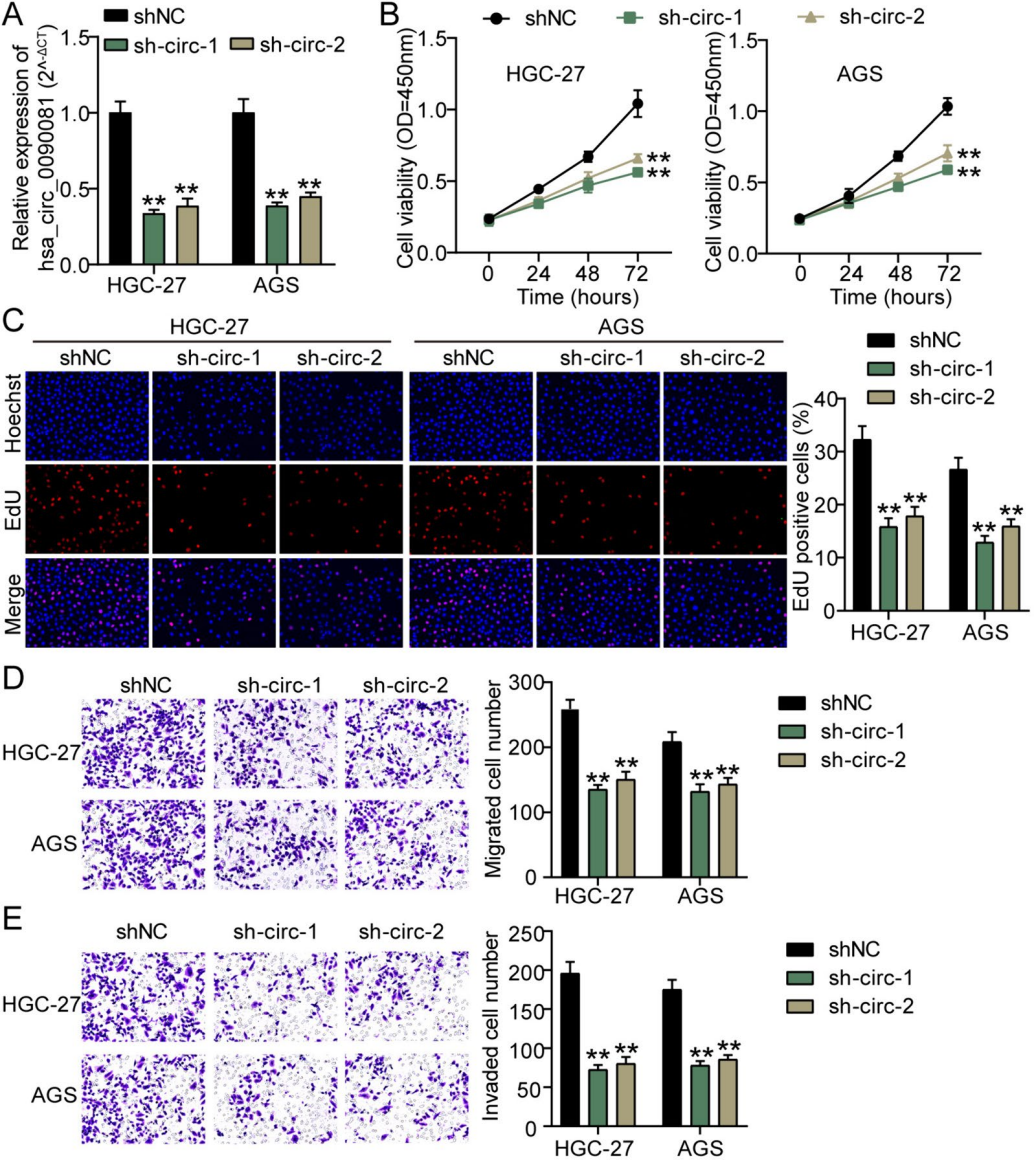
hsa_circ_0090081 silencing curbs GC cells malignancy. **A** qRT-PCR assay showed hsa_circ_0090081 levels in AGS and HGC-27 cells treated with sh-hsa_circ_0090081-1/2 (sh-circ-1/2) or sh-NC. Cell viability, proliferation, migration, and invasion of AGS and HGC-27 cells treated with sh-hsa_circ_0090081-1/2 (sh-circ-1/2) or sh-NC were revealed using **B** CCK-8, **C** EdU, **D** Transwell migration, and **E** Transwell invasion assays, respectively. **P < 0.001 vs. sh-NC

Furthermore, the usefulness of hsa_circ_0090081 in GC was elucidated using transfection of hsa_circ_0090081 siRNA, and data revealed that hsa_circ_0090081 levels in sh-circ-1 and sh-circ-2 were reduced by > 60% and 70%, respectively, compared with sh-NC (Fig. 2A). In CCK-8 and EdU analysis, the viability and proliferation of AGS and HGC-27 cells were restrained via low hsa_circ_0090081 expression (Fig. 2B and C). Furthermore, migration and invasion capacities were revealed using Transwell assays. We observed that low hsa_circ_0090081 expression in AGS and HGC-27 cells showed lower migration and invasion levels (Fig. 2D and E). Thus, hsa_circ_0090081 inhibition prohibits malignancy in vitro of GC cells.

### Upregulated EIF4A3 in GC enrichments hsa_circ_0090081

**Fig. 3:**
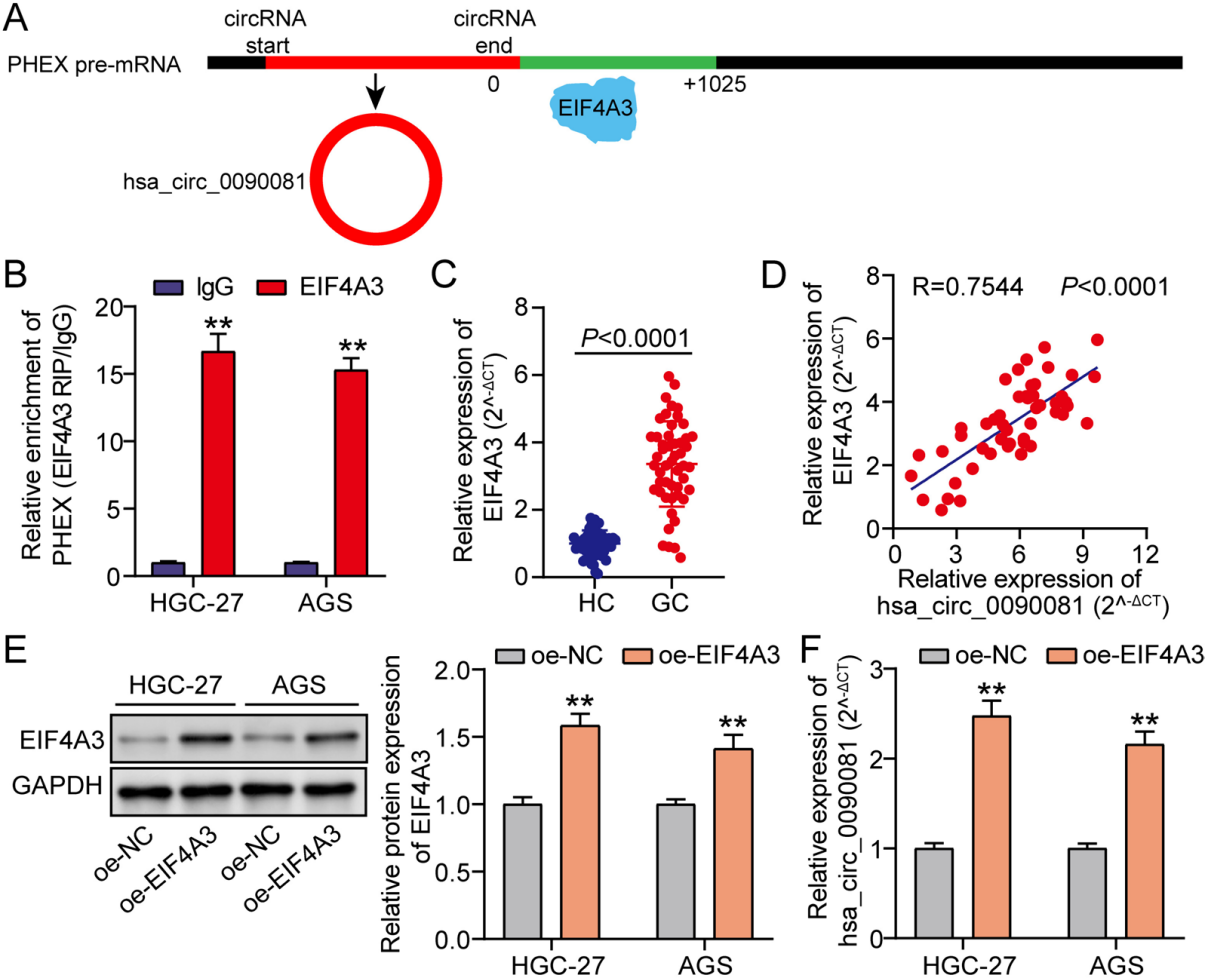
Upregulated EIF4A3 in GC enriched hsa_circ_0090081. **A** circInteractome predicted the binding sites of EIF4A3 on the flanking sequences of the PHEX pre-mRNA transcript. **B** RIP assay revealed the coexistence of PHEX and EIF4A3 in the Ago-2 complex in AGS and HGC-27 cells. **P < 0.001 vs. IgG. **C** qRT-PCR revealed the EIF4A3 levels in human GC and normal samples. **D** Correlation between hsa_circ_0090081 and EIF4A3 in GC tissues. **E** Western blot showed the protein levels of EIF4A3 in AGS and HGC-27 treated with oe-NC and oe- EIF4A3. **P < 0.001 vs. oe-NC. **F** qRT-PCR revealed the hsa_circ_0090081 levels in AGS and HGC-27 treated with oe-NC and oe-EIF4A3. **P < 0.001 vs. oe-NC

The binding sites of EIF4A3 on the PHEX pre-mRNA transcript flanking sequences were predicted using circInteractome (Fig. 3A). Subsequently, their potential interplay in GC cells was analyzed using RIP assays. RIP assay of GC cells validated the coexistence of PHEX and EIF4A3 (Fig. 3B). Additionally, qRT-PCR assay indicated EIF4A3 overexpression in 50 GC samples compared with 50 paired normal samples (Fig. 3C). Interestingly, the positive correlation between EIF4A3 and hsa_circ_0090081 expressions further reinforced their interaction in GC cells (Fig. 3D). Moreover, GC cells were subjected to EIF4A3 overexpression vectors to reveal the function of EIF4A3 on hsa_circ_0090081 levels. EIF4A3 protein was strengthened during EIF4A3 overexpression (Fig. 3E). Moreover, hsa_circ_0090081 levels were also enriched during EIF4A3 enrichment (Fig. 3F). All data indicated that EIF4A3 could bind to PHEX, which enhanced the hsa_circ_0090081 expression.

### hsa_circ_0090081 combines with EIF4A33 to exert its malignant properties in GC

**Fig. 4:**
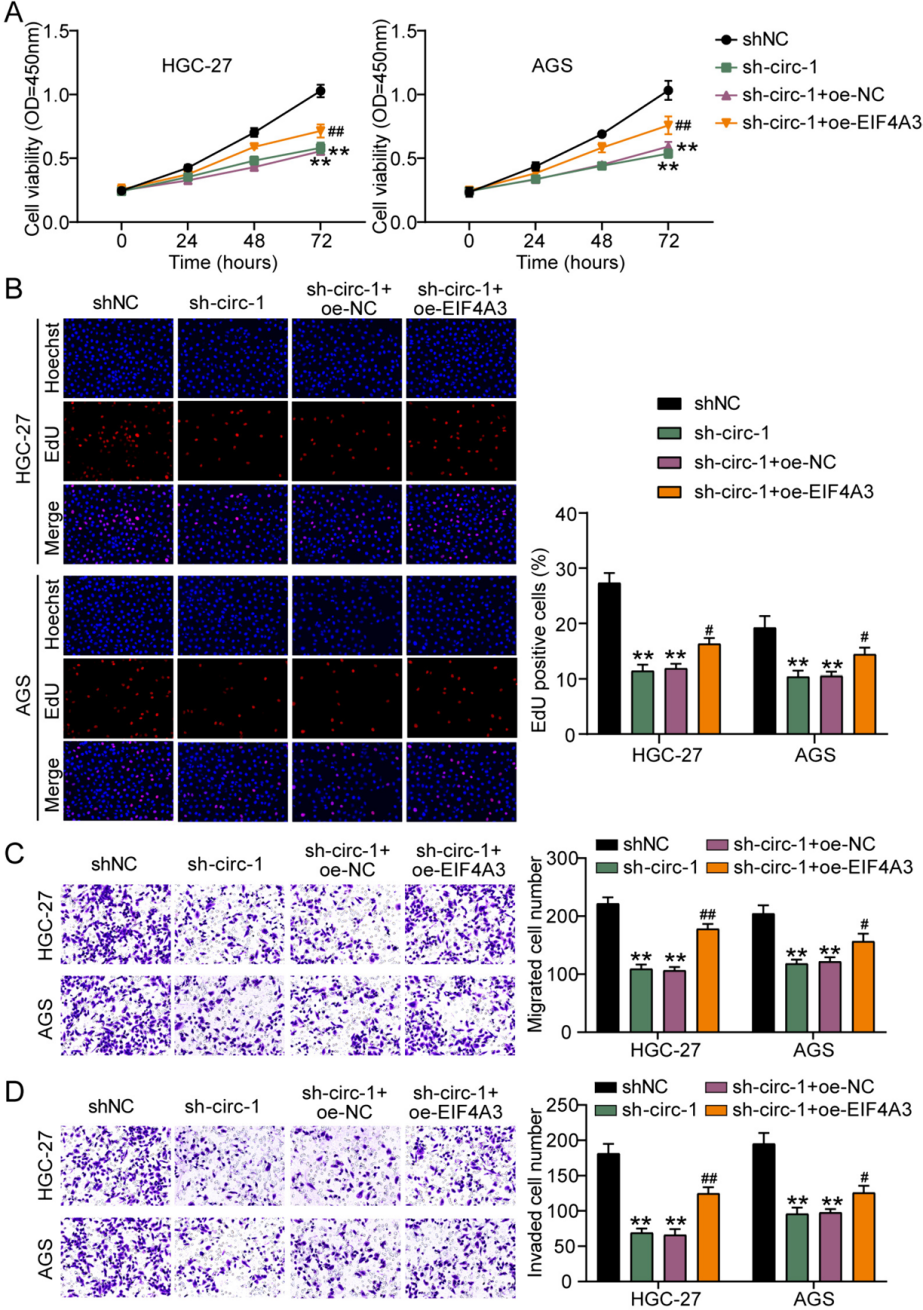
hsa_circ_0090081 combines with EIF4A33 to exert its malignant properties in GC. Cell viability, proliferation, migration, and invasion of AGS and HGC-27 cells treated with EIF4A33 overexpression (EIF4A33), oe-NC, sh-hsa_circ_0090081-1 (sh-circ-1), or sh-NC were revealed using **A** CCK-8, **B** EdU, **C** Transwell migration, and **D** Transwell invasion assays, respectively. **P < 0.001 vs. sh-NC; #P0.05, ##P < 0.001 vs. sh-circ-1 + oe-NC

Serial follow-up evaluations of GC cell manifestations were performed to examine whether the intervention of EIF4A3 with hsa_circ_0090081 affected cellular dysfunction. The sh-circ-1, oe-EIF4A3, sh-NC, and oe-NC were transfected into AGS and HGC-27 cells individually. CCK-8 and EdU outcomes showed that reduced GC cell proliferation caused by hsa_circ_0090081 silencing was nullified by EIF4A3 promotion (Fig. 4A and B). Moreover, EIF4A3 overregulation retrograded the downregulation of cellular metastasis caused by hsa_circ_0090081 inhibition (Fig. 4C and D). Collectively, EIF4A3 regulated hsa_circ_0090081 in GC to promote tumorigenesis.

### EV-delivered hsa_circ_0090081 enhances GC tumorigenesis

**Fig. 5:**
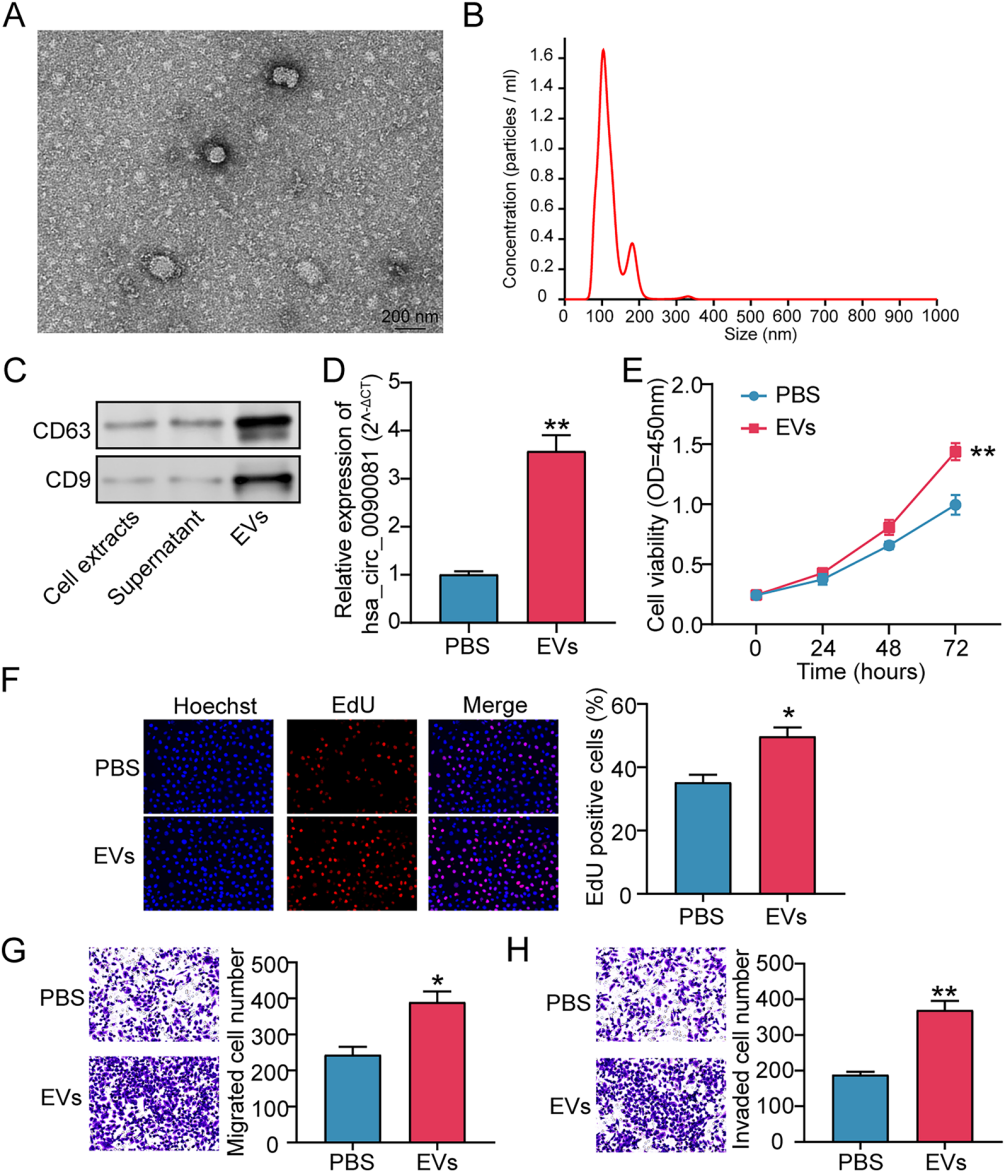
EV-delivered hsa_circ_0090081 enhances GC tumorigenesis. **A** TEM was used to observe the EVs isolated from AGS cells. **B** NTA analyzed the size distribution of EVs from AGS cells. **C** Western blotting analysis of key protein levels of EVs (CD9 and CD63) in EVs, AGS cell extracts, and supernatants. **D** The hsa_circ_0090081 levels in AGS cells treated with EVs or PBS were revealed via qRT-PCR assay. Cell viability, proliferation, migration, and invasion of AGS cells treated with EVs or PBS were revealed using CCK-8 assay (**E**), EdU assay (**F**), Transwell assay (**G**), and Transwell assay (**H**), respectively. *P < 0.05, **P < 0.001 vs. PBS

EVs were exacted in AGS cells and supernatant, and TEM and NTA showed that the diameters of EVs with spherical shapes ranged from 100 to 200 nm (Fig. 5A and B). Subsequently, Western blotting was conducted to clarify key protein levels of EVs (CD9, CD63). Data revealed CD9 and CD63 expressions in EVs, further revealing that EVs were extracted successfully (Fig. 5C). Afterward, EVs were incubated with target AGS cells, and qRT-PCR assay indicated that hsa_circ_0090081 levels were enhanced by over 3.5-fold in target AGS cells incubated with EVs (Fig. 5D). Data manifested increased cell proliferation in EV groups compared with PBS (Fig. 5E and F). Subsequently, cell migratory ability and invasiveness were significantly increased in EV versus PBS groups (Fig. 5G and H). Thus, GC cell-derived EVs transferred hsa_circ_0090081 to facilitate GC tumorigenesis.

## Discussion

Epidemiological studies have highlighted that the occurrence of GC is gradually increasing in young individuals [19]. Furthermore, EV-delivered circRNA therapy has been investigated as an emerging technology in GC treatment. Here, we identified a critical role for hsa_circ_0090081, regulated by EIF4A3, to be carried by EVs in GC progression. Moreover, hsa_circ_0090081 is upregulated in GC, and silencing inhibits the ability of GC cells to survive, migrate, and invade in vitro. Additionally, this study identified EIF4A3 as an upstream regulator of hsa_circ_0090081, positively regulating hsa_circ_0090081 expression. Subsequently, hsa_circ_0090081 was upregulated in GC cell EVs, and malignancy was promoted in vitro after target GC cells were cocultured with EVs. These findings showed a novel regulatory network during GC development.

The regulatory role of circRNAs in GC has been widely reported. Yuan et al. [20] found that circRNA_102231 levels were upregulated in GC plasma samples and tissues, which could act as biomarkers for GC prognosis and diagnosis. Ma et al. [21] revealed that the significant hsa_circ_0004872 downregulation in GC tissues was associated with local lymph node metastasis and tumor size, and forced hsa_circ_0004872 levels restrained GC cell malignancy and tumorigenicity. Zhang et al. [22] found low circDIDO1 expression in GC, and its inhibition was linked with poor prognosis, distal metastasis, and its overexpression reduced GC growth and metastasis. For the first time, hsa_circ_0090081 was enriched in GC and associated with shorter patient survival. Additionally, hsa_circ_0090081 silencing reduced GC cell invasion, migration, and survival in vitro, indicating that hsa_circ_0090081 plays a role as a procancer factor in GC.

RNA-binding proteins (RBPs) are implicated in all aspects of the circRNA life cycle, including execution of functions, post-transcriptional regulation, and production [23]. Several studies have shown that the interaction between circRNAs and RBPs has crucial implications for tumors and other diseases and may serve as a biomarker of disease [24]. EIF4A3 could act as an RBP because it binds to a wide range of RNAs and exerts carcinogenesis effects. For example, EIF4A3 binds to the reverse splice junction and downstream flanking sequences of circARHGAP29 to induce cyclization and cytoplasmic export of circARHGAP29, thereby promoting aerobic glycolysis in doxorubicin-resistant prostate cancers [11]. EIF4A3 directly binds to circABCA5 in GC to promote its stability and expression, accelerating cancer progression [25]. EIF4A3 inhibits circRNA_100290 formation by binding to its flanking site to promote epithelial–mesenchymal transformation and invasion and survival of GC cells [26]. This study revealed that EIF4A3 was an upstream regulator of hsa_circ_0090081 and promoted stable hsa_circ_0090081 formation. Additionally, EIF4A3 overexpression promoted GC cell growth, invasion, migration, and partial elimination of the repressive effect of hsa_circ_0090081 silencing on GC cell malignancy. Thus, EIF4A3 accelerates GC malignancy by upregulating hsa_circ_0090081.

EVs play a crucial role in tumor progression as a new means of intercellular information transfer [27]. CircSTAU2 could be encapsulated in EVs and delivered to receptor GC cells, thereby promoting GC tumorigenicity [28]. High EV-delivered circ_0063526 expression in serum is associated with adverse responses to the chemotherapy treatment of patients with GC. Knockdown of EV-delivered circ_0063526 suppresses chemoresistance by inhibiting metastasis and autophagy in GC cells [29]. We identified a novel EV delivery mechanism in GC, wherein hsa_circ_0090081 was encapsulated in GC cell EVs for delivery to target GC cells, resulting in enhanced invasion, migration, and proliferation of target GC cells in vitro, indicating that EV delivery of hsa_circ_0090081 enhances GC progression.

This study has limitations. First, the effect of EV delivery of hsa_circ_0090081 in GC cells should be explored more by modulating EV-delivered hsa_circ_0090081 levels. Subsequently, the downstream mechanism of hsa_circ_0090081 in GC should also be further explored. In addition, the association of hsa_circ_0090081 with clinical features should be investigated by collecting more clinical samples. Furthermore, this study used a circRNA microarray including three samples per group from a public database GEO DatesSets, which may limit other circRNAs who may play key roles in GC. Thus, we will perform microarray analysis using our collected clinical samples.

In summary, we showed the novel circRNA, hsa_circ_0090081, was positively regulated by EIF4A3 and delivered by EVs to promote GC proliferation, invasion, and migration. Our results may help in formulating rational therapeutic strategies in GC.

## Methods

### Tissue specimens

At Wuhan Third Hospital, patients with GC who underwent surgery donated 50 pairs of GC tissues and adjoining healthy tissues. All subjects provided an informed consent form before sample acquisition. The samples were cryopreserved at − 80 ℃ for later use. Table 1 summarizes the clinicopathological features of all patients with GC. The ethics committee of Wuhan Third Hospital approved this study.

**Table 1:** The clinical characteristics of the gastric cancer patients

Characteristics	Total = 50	Percentage (%)
Age (years)		
≤ 60	17	34.0
> 60	33	66.0
Sex		
Male	34	68.0
Female	16	32.0
Tumor size (cm)		
≤ 1	6	12.0
1–2	13	26.0
> 2	31	62.0
TNM stage		
I–II	21	45.7
III–IV	29	54.3
Differentiation		
Well	6	12.0
Moderate	21	42.0
Poor	23	46.0
Lymphovascular invasion		
Yes	27	54.0
No	23	46.0
Lymph node metastasis		
Yes	31	62.0
No	19	38.0

### Cell culturing

The GC cell lines comprising AGS (BNCC338141) and HGC-27 (BNCC338546) were provided by China, BeNa Culture Collection. Additionally, AGS cells in the F-12 K medium with 10% FBS and HGC-27 cells in RPMI-1640 medium with 10% FBS were incubated in an incubator with 5% CO_2_ and a temperature of 37 ℃. The medium and FBS were sourced from Huayun Biotech., China.

### Cell nucleus/cytoplasm fraction isolation

The cell nucleus and cytoplasm were fractionated using nuclear and cytoplasmic extraction reagents (NobleRyder, China) as instructed before qRT-PCR analysis of hsa_circ_0090081 in AGS and HGC-27. GAPDH and U6 served as the cytoplasmic and nuclear controls, respectively.

### RNase A treatment

Total RNA from AGS and HGC-27 were digested with RNase R (Mock) at 37 ℃. After 30 min, qRT-PCR was used to determine the levels of hsa_circ_0090081 and PHEX (linear of hsa_circ_0090081) with normalization to Mock in cells.

### qRT-PCR

Following the manufacturer’s protocol, the entire RNA was obtained through Trizol reagent (General Biotech., China). Subsequently, cDNA was synthesized from a 1 μg RNA by using the First Strand cDNA Synthesis Kit (Shanghai Yubo, China). Afterward, qPCR was conducted using SYBR Green qPCR Mastermix (Qiangen, Germany). By using the 2^−ΔΔCt^ method, we determine the relative hsa_circ_0090081 and EIF4A3 expression via GAPDH as the internal reference. Table 2 shows the primers.

**Table 2:** Sequence of primers for qRT-PCR

Gene	Primer type	Sequence
hsa_circ_0090081	Forward	5′-AGCTTTGTGGGAAGCCTAAA-3′
	Reverse	5′-TTCATGTCCGACAATTACTCC-3′
EIF4A3	Forward	5′-CAACGAGCAATCAAGCAG-3′
	Reverse	5′-GTGGGAGCCAAGATCAAA-3′
GAPDH	Forward	5′- GACCCCTTCATTGACCTCAAC-3′
	Reverse	5′-CGCTCCTGGAAGATGGTGAT-3′

### Cell transfection

The nontargeting shRNAs (negative control, sh-NC) and shRNAs of hsa_circ_0090081 (sh-circ-1/2) were purchased from Ribobio (China). The pcDNA3.1 used for EIF4A3 overexpression vectors (oe-EIF4A3) and its empty vector (oe-NC) was also purchased from Ribobio. Cell transfection was conducted using a liposomal transfection reagent (Yeasen, China). The transfection of pcDNA (2 μg/mL) or siRNA (50 nM) into AGS and HGC-27 cells was conducted after the cells reached 60% confluence. After 48-h transfection (5% CO_2,_ 37 ℃), transfection efficiency was examined using qRT-PCR.

### EVs isolation, identification, and coculture

Briefly, AGS cells and culture medium were gathered and centrifuged at 4 ℃ [10 min, 300*g*] to dislodge cell pellets. Supernatants were collected and centrifuged at 2000 ×*g* for 10 min to dislodge dead cells, at 10,000 ×*g* for another 10 min to dislodge cell debris, and finally at 110,000 ×*g* for 2 h, and the pellet was collected as EV. The isolated EVs were resuspended in PBS, incubated with 500-µL total exosome isolation reagent, and purified using Total Exosome Isolation Kit (4,478,359, Invitrogen, USA). After vortexing, the isolated EVs were incubated at 4 ℃ overnight. After a day, the isolated EVs were centrifuged for 1 h at 10,000 ×*g* at 4 ℃. After discarding the supernatant, the purified EVs at the bottom of the tube were collected and resuspended in PBS. The isolated EVs were identified using transmission electronic microscopy (TEM), and size distribution and EV concentration were determined using nanoparticle tracking analysis (NTA)\, and key protein levels of EVs (CD9, CD63) were detected using Western blot. For the coculture, the collected EV precipitates were resuspended in precooled PBS. Subsequently, 40-µL EV (concentration: 1.3 × 10^8^ particles/mL) or an equal volume of PBS was incubated with 2 × 10^5^/well of AGS cells in six-well plates for 24 h based on a previous study [18].

### CCK-8 assay

AGS and HGC-27 cells (5000 cells/well) were routinely cultured using a 96-well plate for specified time points (0, 24, 48, or 72 h). Subsequently, the cells were subjected to treatment with CCK-8 (Beyotime, China) for 2 h. A microplate reader (Molecular Devices, China) was employed to measure the optical density of these cells at 450 nm.

### EdU assay

Cell proliferation ability was evaluated using the EdU cell proliferation kit (C0071S, Beyotime). Briefly, cultured AGS and HGC-27 cells (1 × 10^6^ cells/mL) were integrated into EdU solution incubated with 4% formalin fixation for 30 min, followed by staining with 1 × Apollo staining solution for 30 min, and incubation with Hoechst for 30 min. To clarify cell proliferation, the EdU-positive cell rates were analyzed under a fluorescence microscope (Olympus, Japan).

### Transwell assay

AGS and HGC-27 cells (2 × 10^4^ cells/well) were resuspended in DMEM medium (serum-free, 500 µL) and infused into the upper chamber with the presence or absence of Matrigel to evaluate migration and invasiveness. Meanwhile, neither compartment was infused with medium (plus 10% FBS, 750 µL). After 24-h cultivation, 0.5% crystal violet was added for cell staining. The Olympus light microscope (Japan) was used to photograph three randomly selected fields.

### Western blotting

Transfected AGS and HGC-27 cells were lysed in RIPA (Solarbio, China). Proteins were separated using SDS-PAGE (10%) and electrically moved to PVDF membranes, which were inversed at 25 ℃ for 1 h in 5% nonfat milk and probed with anti-GAPDH (ab9485), anti-EIF4A3 (ab180573), anti-CD63 (ab134045), and anti-CD9 (ab134079) at 4 ℃. After 24 h, the blots were mixed with HRP (ab288151) secondary antibody (1 h, 37 ℃). The antibodies were obtained from Abcam, USA. Immunoreactive proteins were visualized using BeyoECL Plus (P0018S, Beyotime).

### RIP assays

The RIP kit (BersinBio, China) was used to detect PHEX expression. AGS and HGC-27 cells were mixed with RIP buffer. Magnetic beads coated with IgG or EIF4A3 antibodies were mixed into the lysate. The abundance of PHEX pre-mRNA in RIP-complex was determined using qRT-PCR.

### Statistical analysis

Using Prism 8.0.1 (GraphPad, USA), data from three replicated experiments were examined and presented as mean ± standard deviation. Statistical significance was determined at P < 0.05, and data were analyzed using Student’s t-test (two groups) and analysis of variance (several groups). The relevance of hsa_circ_0090081 to EIF4A3 was analyzed using Pearson’s analysis. Log-rank (Mantel–Cox) test was performed to clarify the effect of hsa_circ_0090081 on the prognosis of patients with GC.

### Supplementary Information

**Additional file 1.**

## Data Availability

All data that had been produced and/or analyzed in the duration of this research have been appended in this manuscript.

## Abbreviations

GC: Gastric cancer
CircRNA: Circular RNA
EIF4A3: Eukaryotic translation initiation factor 4A3
