# Supplementary figures and images for: Extracellular vesicle-delivered hsa_circ_0090081 regulated by EIF4A3 enhances gastric cancer tumorigenesis
Source: Cell Div. 2024 Jun 11;19:19. doi: 10.1186/s13008-024-00123-z (PMC11165812; doi:10.1186/s13008-024-00123-z)

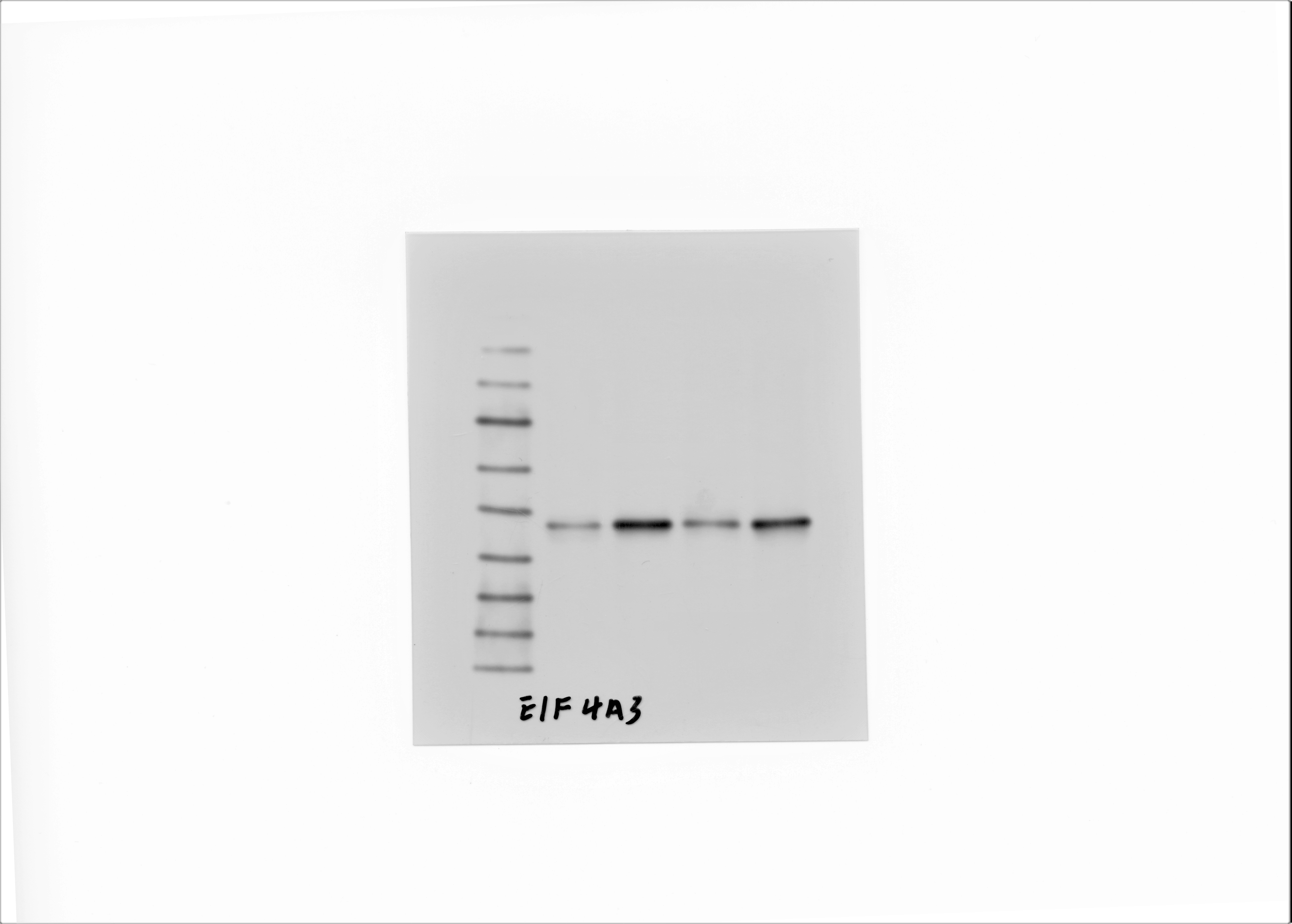

Supplement: Supplementary file 1 — Additional file 1. [file 13008_2024_123_MOESM1_ESM.zip › western blot-revised/Fig.3E EIF4A3.tif]

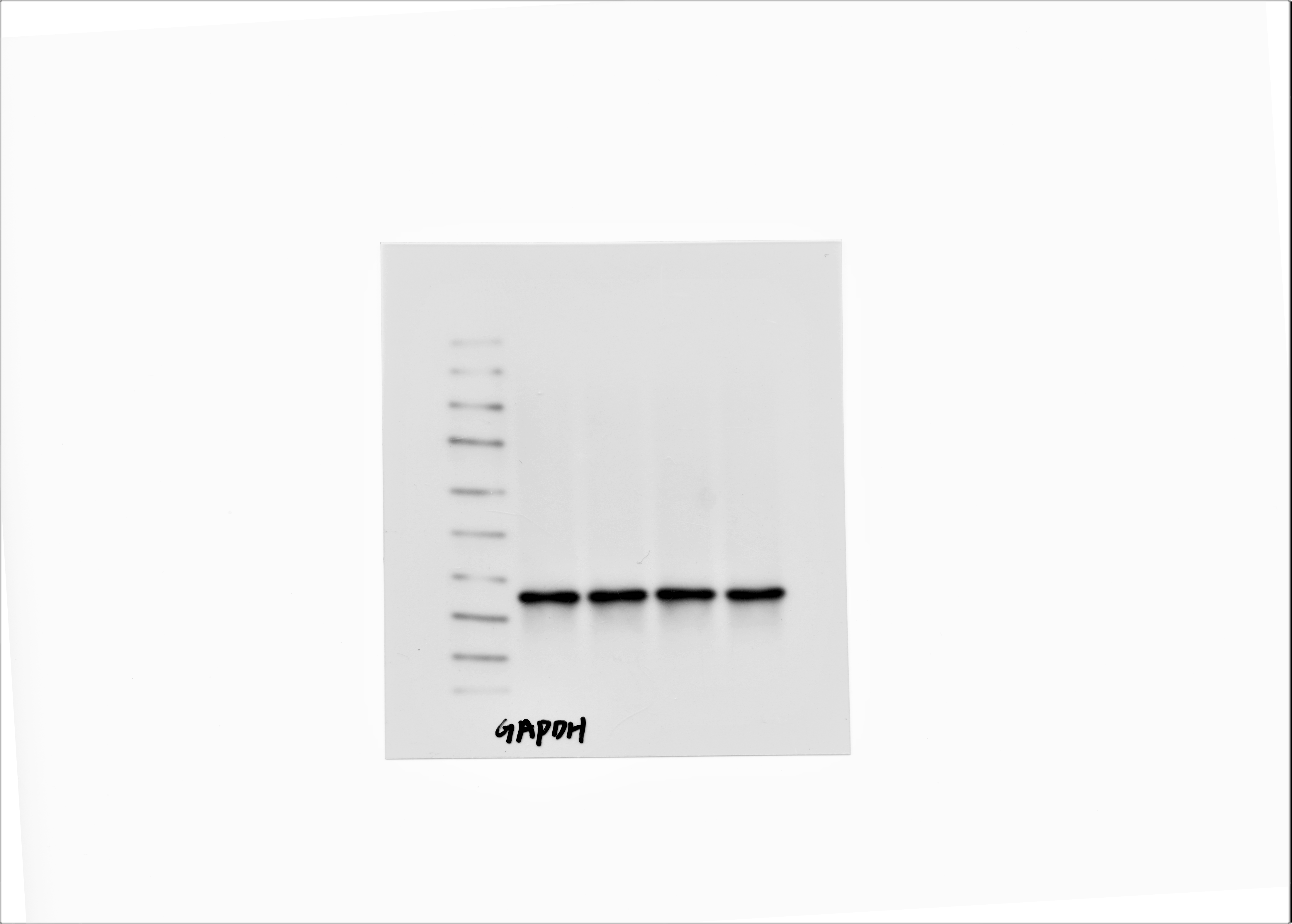

Supplement: Supplementary file 1 — Additional file 1. [file 13008_2024_123_MOESM1_ESM.zip › western blot-revised/Fig.3E GAPDH.tif]

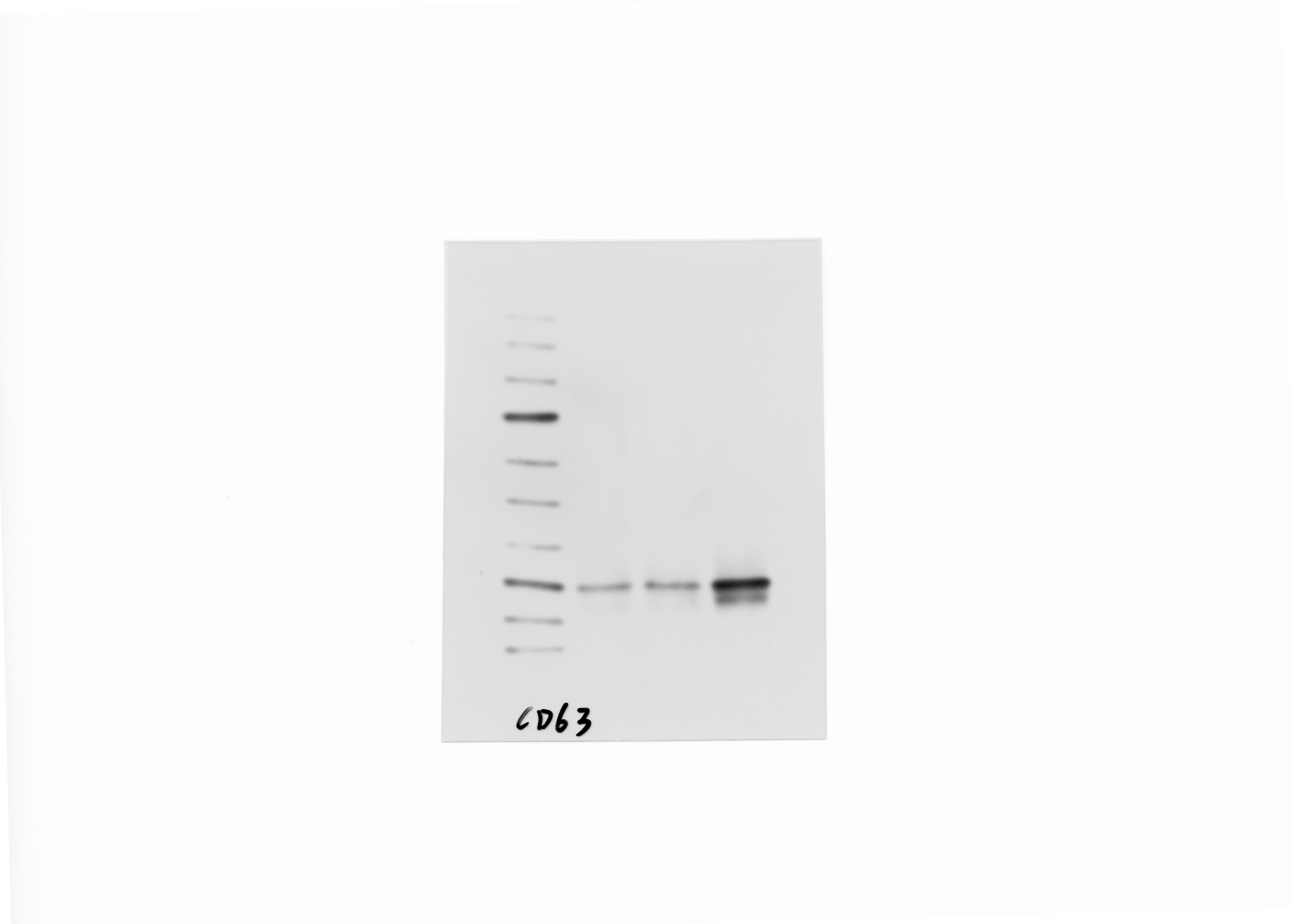

Supplement: Supplementary file 1 — Additional file 1. [file 13008_2024_123_MOESM1_ESM.zip › western blot-revised/Fig.5A CD63.tif]

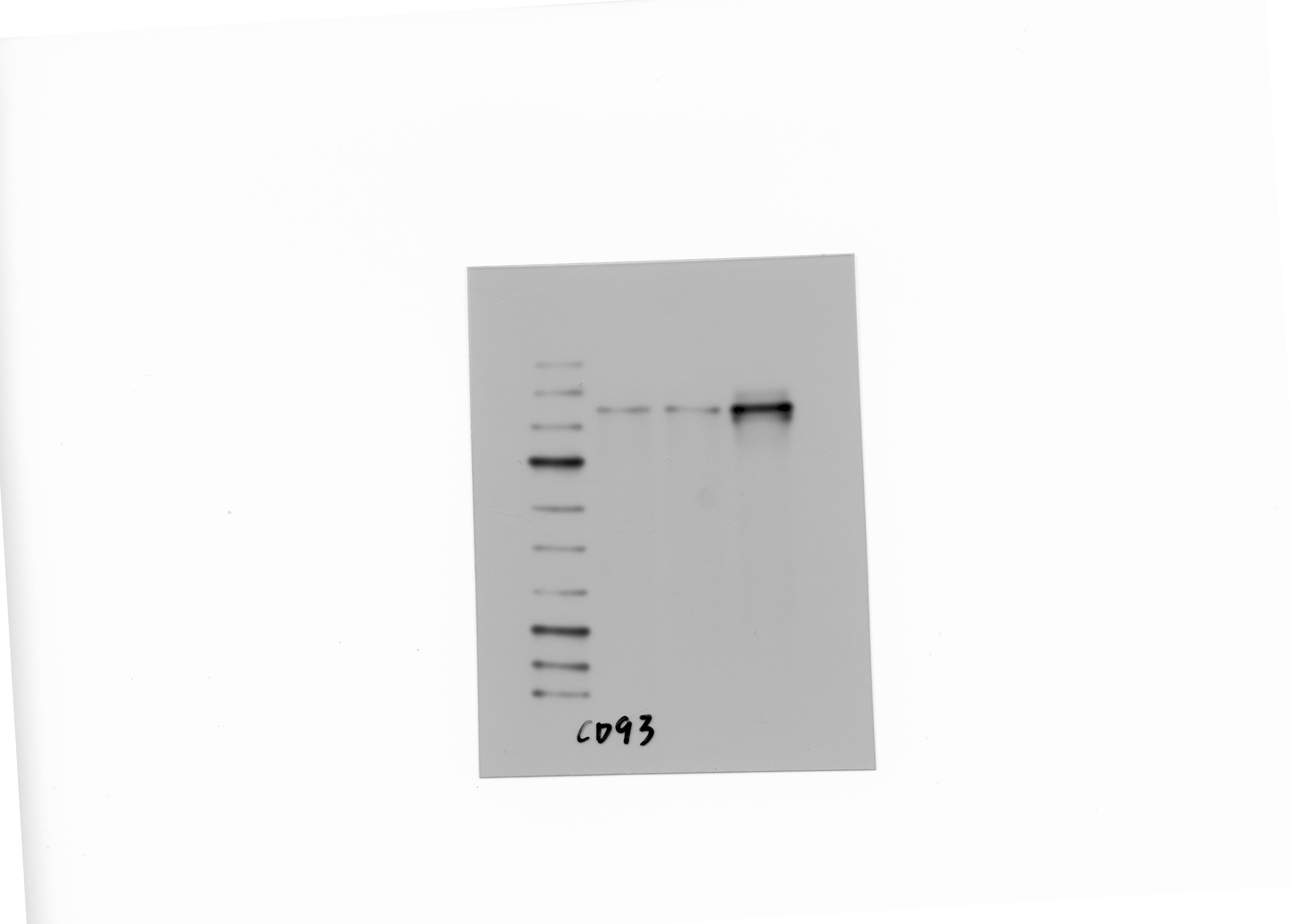

Supplement: Supplementary file 1 — Additional file 1. [file 13008_2024_123_MOESM1_ESM.zip › western blot-revised/Fig.5A CD93.tif]
